# The essential role of the redox balance in adrenal steroidogenesis

**DOI:** 10.20945/2359-4292-2026-0049

**Published:** 2026-05-15

**Authors:** Juliana Lourenço Gebenlian, Aline Faccioli Bodoni, Fernanda Borchers Coeli-Lacchini, Margaret de Castro, Sonir Roberto Rauber Antonini

**Affiliations:** 1 Departamento de Pediatria, Faculdade de Medicina de Ribeirão Preto, Universidade de São Paulo, Ribeirão Preto, SP, Brasil; 2 Departamento de Clínica Médica, Faculdade de Medicina de Ribeirão Preto, Universidade de São Paulo, Ribeirão Preto, SP, Brasil

**Keywords:** Adrenal steroidogeneses, oxidative stress, *NNT* gene

## Abstract

**Objective:**

To analyze the impact of nicotinamide nucleotide transidrogenase (NNT) enzyme impairment on cortisol production, reactive oxygen species (ROS) formation, mitochondrial activity, and mitogen-activated protein kinase 8 (MAPK8) expression in the adrenocortical cell line.

**Materials and methods:**

siRNA NNT knockdown was performed in H295R adrenocortical cells to evaluate the effects on cortisol secretion (RIA) and reactive oxygen species (ROS) intracellular production (DCFDA) in basal conditions and after stimulation with 10 uM forskolin. MAPK8 mRNA relative expression and protein localization were evaluated using quantitative PCR (qPCR) and immunofluorescence, respectively.

**Results:**

NNT knockdown in the H295R adrenal cells specifically reduced NNT RNA and protein levels. Under basal conditions, no changes in ROS intracellular production or cortisol secretion were seen in NNT-depleted H295R cells. However, under forskolin (10 µM), a potent steroidogenesis agent, we observed a marked reduction in cortisol production (p < 0.0001) at the expense of normal ROS intracellular production and increased MAPK8 expression (p = 0.008).

**Conclusion:**

NNT-deficient adrenocortical cells can maintain mitochondrial homeostasis and cortisol secretion in basal conditions. However, under stress, these cells seem to maintain the redox balance at the expense of impaired steroidogenesis. MAPK8 pathway activation appears to compensate for NNT deficiency.

## INTRODUCTION

Cortisol production occurs partially within mitochondria, resulting in the release of reactive oxygen species (ROS). ROS must be actively eliminated to maintain homeostasis in the intramitochondrial environment. For this reason, adrenal cortex cells are supplied with high levels of enzymatic and non-enzymatic antioxidants to maintain redox homeostasis. Disturbances in the redox homeostasis in the intramitochondrial environment of adrenal cells may have a deleterious effect on steroidogenesis ^(1)^.

The maintenance of redox homeostasis, which reflects the delicate balance between ROS and antioxidant systems, plays a crucial role in hormonal regulation and cellular function. Some hormones, such as melatonin, estrogens, insulin, and glucagon, possess antioxidant properties, protecting cells from oxidative damage and modulating essential physiological processes, including development, growth, and aging. Conversely, hormones such as catecholamines, thyroid hormones, and corticosteroids can increase ROS production, contributing to oxidative stress. Alterations in hormonal balance or endocrine system function can modify redox states in vertebrates, leading to pathological conditions associated with oxidative stress ^(2)^.

Abnormal oxidative stress is related to a multitude of pathological conditions, including neurodegenerative disorders, diabetes mellitus, cardiovascular disease, aging, and changes in hormone synthesis by the adrenal glands. The effects of oxidative stress or ROS accumulation on steroidogenesis are partially understood. However, the mechanisms involved have not yet been determined ^(3,4)^.

The antioxidant defense system has complex and specific mechanisms for each cell type. Tissues with greater energy consumption, such as the liver, brain, and steroidogenic tissues, are supplied with high levels of enzymatic and non-enzymatic antioxidants, such as superoxide dismutase (SODs), glutathione reductase, glutathione peroxidase (GPX), and catalase. These antioxidant components are essential for the detoxification of ROS ^(1)^. The glutathione cycle detoxification system depends on nicotinamide adenine dinucleotide phosphate (NADPH), which is synthesized in the intramitochondrial environment through the enzyme Nicotinamide Nucleotide Transhydrogenase (NNT) ^(5)^.

The NNT protein is crucial for the elimination of ROS and the maintenance of cellular homeostasis. NNT requires the energy of the mitochondrial proton gradient to produce high concentrations of NADPH, which is responsible for regenerating reduced glutathione (GSH) into oxidized glutathione (GSSG), thereby maintaining a high GSH/GSSG ratio ^(5)^.

Loss-of-function mutations in the NNT gene have been described in patients with primary adrenal insufficiency, most of which are associated with the familial glucocorticoid deficiency (FGD) phenotype ^(6,7)^.

In the human adrenocortical carcinoma cell line (H295R), silencing the NNT gene through small interfering RNA (siRNA) reduced the GSH/GSSG ratio and promoted an increase in O2- concentrations. Such effects demonstrate the dysregulation of redox potential promoted by decreased NNT activity ^(6)^.

In a previous study, we developed a knockout model for NNT using H295R cells and CRISPR/Cas9 technology. In that study, we observed that NNT impairment resulted in increased ROS production, decreased mitochondrial labeling, and a decrease in the size and density of cholesterol droplets. A reduction in the secretion of cortisol and aldosterone was also observed. These data indicate that the abolition of NNT generates a state of increased oxidative stress, in addition to inhibiting steroidogenesis in affected cells ^(8)^.

In the C57BL6/J mouse lineage, which is naturally deficient in NNT, a lack of organization in the zona fasciculata of the adrenal gland is observed during embryological development. A reduction in corticosterone secretion and expression of the CYP11A1 gene, which participates in the mitochondrial stage of adrenal steroidogenesis ^(6,9)^, was also noted. Additionally, knockout mice for the NNT gene, when subjected to stress, exhibited impairment in the stimulated increase in corticosterone secretion and greater ACTH secretion compared to control animals. An increase in plasma concentrations of neurotransmitters such as norepinephrine and dopamine was also noted ^(10)^.

Decreased activity of NNT, in adrenal pheochromocytoma cells (PC12 cell line), reduced intracellular NADPH levels, reduced the GSH/GSSG ratio, and increased H2O2 levels, culminating in an increase in the oxidative state, as also observed in H295R cell line. The reduction in NNT expression in these cells also resulted in an increased expression of phosphorylated mitogen-activated protein kinase 8 (MAPK8) ^(11)^. These studies demonstrate the important role of MAPK8 and raise the question about its role in maintaining the adrenal cells’ redox state and a possible crosstalk with the NNT enzyme.

In the present study, our objective was to evaluate the effects on steroidogenesis in the case of changes in antioxidant defense mechanisms. In H295R human adrenocortical tumor cells, we investigated the effects of reducing NNT expression on steroidogenesis under basal conditions and in response to induced stress. In addition, considering the established role of MAPK signaling in cellular stress responses, we investigated MAPK8 as a potential compensatory mechanism contributing to mitochondrial homeostasis and redox balance. These studies provide a better understanding of the oxidative stress affected by adrenal function.

## MATERIALS AND METHODS

### H295R cell culture

H295R adrenocortical carcinoma cell line (RRID: CVCL_0458) was grown in DMEM F12 Medium supplemented with 5% NuSerum, 1% penicillin/streptomycin solution, and insulin-transferrin-selenium in a humidified incubator at 37 °C in a 5% CO_2_ atmosphere. The H295R cell line was obtained from the American Type Culture Collection (ATCC).

The medium was replaced with fresh medium containing either vehicle or test drugs such as forskolin (FSK; Merck-Aldrich) (final concentration = 10 µM) and hydrogen peroxide (H_2_O_2_; Merck-Aldrich) (final concentration = 1 mM). Forskolin treatment was performed for 24 h, and H_2_O_2_ treatment for 3 h. Cells were maintained in complete medium and were not subjected to serum starvation. All the stock solutions of the test drugs were prepared in DMSO (Merck-Aldrich) or water, respectively. Within the experiment, the total final concentration of DMSO (less than 0.1%) was maintained constant across conditions.

### siRNA transfection - *NNT* silencing

The cells were seeded in 96-well culture plates, for evaluation of viability, 24 for mRNA relative expression, and immunofluorescence or 6-well culture plates for protein expression analysis. After 24 hours, the medium was replaced by antibiotic-free complete medium, and the cells were transfected with 40 nM of either small-interfering RNA (siNNT - L-009809-00-0005) or silencer negative control (NT - siNonTargeting D-001206-13-05) (SMARTpool ON-TARGET plus SiRNA, Horizon) using DharmaFECT 1 transfection reagent (Horizon). After 24 hours of incubation, the medium was replaced, and the cells were harvested after 48 hours for gene expression. Two independent experiments were performed in triplicate.

### Viability

Cells (3 x 10^4^ per well) were seeded in 96-well plates. After 96 h, Cell Titer 96 Aqueous One Solution (Promega) was added to each well, which was followed by incubation for 1 h. Absorbance at 490 nm was read with a microplate reader (Bio-Rad).

### RNA isolation and RT-qPCR

Cells (3 x 10^5^ per well) were seeded in 24-well plates. Total RNA was extracted by using the TRIzol Reagent (Thermo Scientific), and mRNA was subjected to reverse transcription from 500 ng of total RNA by using the High-Capacity cDNA Reverse 260 Transcription kit and MultiScribe enzyme (Thermo Scientific). To assess gene expression using TaqMan assays (Thermo Scientific), quantitative Real-Time PCR (qPCR) of the following genes was performed: NNT (Hs00966097_m1), *CYP11B1* (HS01596404), *CYP11A1* (HS00897320_M1), and *GUSB* (4326320E), *ACTB* (4352935) were used as an endogenous control. We performed two independent experiments in triplicate for the final analyses. For all the analyses , mRNA relative expression value was determined by the 2^-∆∆Ct^ method. Results were normalized to the Ct values of the endogenous controls *GUSB* and *ACTB*.

### Western blot analysis

Cells (1 x 10^6^ per well) were seeded in 6-well plates. Cells were lysed with the CelLytic M (Merck-Aldrich), and Protease and Phosphatase Inhibitor Cocktails (P8340 and P5726, both Merck-Aldrich) were added.

Protein concentration was measured by the Pierce™ BCA Protein Assay Kits (Thermo Scientific). Equal amounts (30 µg) of protein were subjected to SDS-PAGE, transferred to nitrocellulose membranes, blocked in TBST-T containing 5% Blotting-Grade Blocker (Bio-Rad), and probed with the aforementioned anti-NNT antibody (dilution: 1:200; RRID: AB1079495). Anti-GAPDH antibody (dilution: 1:1,000; RRID: AB_627678) was used as a loading control. Immunocomplexes were visualized with horseradish peroxidase (HRP) conjugated anti-mouse (dilution: 1:4,000, RRID: AB_631736) and anti-rabbit (dilution: 1:3000; RRID: AB_628497) antibody and developed with enhanced chemiluminescence Kit ECL Western Blotting Detection Reagents (GE Healthcare) on a ChemiDoc XRS+ System (Bio-Rad, Hercules, CA, USA). Acquired bands were analyzed using the Image Lab™ software (Bio-Rad).

### Mitochondrial function analysis

A parameter of mitochondrial function was described previously ^(12)^. Intracellular reactive oxygen species (ROS) levels; 20,70-dichlorodihydrofluorescein diacetate (H2DCFDA - (Thermo Scientific) was used. Cells were incubated with 5 µM H2DCFDA at 37 °C for 30 min, washed, and analyzed by using the BD-FACS Canto flow cytometer (Becton Dickinson); 495-nm excitation and 527-nm emission wavelengths were used. Mean fluorescence intensities were analyzed with the BD - FACS-Diva Version 6.1.3 software (Becton Dickinson). At least 10,000 cells were analyzed.

MitoTracker Deep Red FM (Thermo Fisher Scientific), a dye that stains mitochondria in live cells, was used to measure mitochondrial intensity. Cells were incubated with MitoTracker Deep Red (250nM) or DAPI (0.5 µg/mL) at 37 °C for 30 min. The cells were fixed, and mounting medium (Thermo Fisher Scientific, MA, USA) was added. Digital images were acquired with the Confocal Microscope Leica SP5 (Leica Microsystems). The different fluorophores were excited using the 644-nm, 665-nm, 365-nm, and 405 nm wavelengths. LAS AF version 2.7.3.9723 software (Leica Microsystems) was used for acquisition. Mean fluorescence intensities were analyzed with the BD - FACS-Diva Version 6.1.3 software (Becton Dickinson) using 644-nm excitation and 665-nm emission wavelengths. Two independent experiments were carried out in triplicate.

Intracellular reactive oxygen species (ROS) levels were analyzed using the 20,70-dichlorodihydrofluorescein diacetate (H2DCFDA; Thermo Scientific). Cells were incubated with 5 µM H2DCFDA at 37 °C for 30 min and analyzed in the BD-FACS Canto flow cytometer (Becton Dickinson), using 495-nm excitation and 527-nm emission wavelengths. Mean fluorescence intensities were analyzed with the BD - FACS-Diva Version 6.1.3 software (Becton Dickinson). Two independent experiments were carried out in triplicate.

### Cortisol assay

Cortisol was measured by using an in-house developed radioimmunoassay (RIA) as described previously ^(13)^.

### Immunofluorescence

Cells (3 x 10^5^ per well) were seeded on coverslips in 24-well plates. After 24 hours, the cells were fixed in 4% formaldehyde and blocked with 1% BSA. MAPK8 was detected using primary antibody for MAPK8 protein ((dilution: 1:200) AB18680), and Donkey anti-Rabbit IgG (H+L) Highly Cross-Adsorbed Secondary Antibody, Alexa Fluor™ 488 as secondary antibody (RRID:AB_2535792) Nuclear staining was accomplished using DAPI (dilution: 1:25000 #4083, Cell Signaling Technology), and the slides were set with ProLong™ Diamond (Thermo Fisher Scientific). Fluorescence was acquired with the Confocal Microscope Leica SP5 (Leica Microsystems). The different fluorophores were excited at 644-nm, 665-nm, 365-nm, and 405-nm.

### Statistical analysis

Data are presented as the mean ± SD or the mean ± SEM. Statistical significance was determined by using a Student’s t-test or one-way ANOVA followed by Tukey’s. The GraphPad Prism 8.0^®^ software was used for these analyses (GraphPad, San Diego, CA, USA), and the level of significance was set at p ≤ 0.05.

## RESULTS

### Relationship between oxidative stress and cortisol production

**Figure 1:**
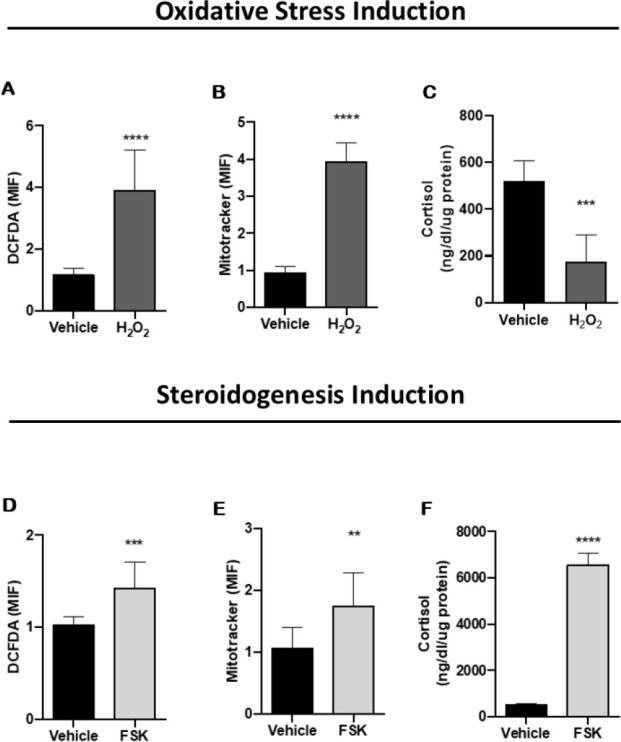
Relationship between oxidative stress and cortisol production. (**A**) Reactive oxygen species (ROS) production, quantified with 20,70-dichlorodihydrofluorescein diacetate (DCFDA, Merck-Aldrich), cells treated with hydrogen peroxide (H_2_O_2_) show an increase in ROS production when compared to the vehicle (n=6; p≤ 0.0001). (**B**) Cells labeled with Mitotracker Red show an increase in mitochondrial activity after treatment with H_2_O_2_ (n=6; p≤ 0.0001). (**C**) Reduction in cortisol production after induction of oxidative stress (n=6; p=0.0004). (**D**) Increase in ROS production after steroidogenesis induction with forskolin (FSK) treatment (n=6; p= 0.0002). (**E**) Important increase of the mitochondrial activity in H295R cells after steroidogenesis induction (n=6; p=0.006). (**F**) Increased cortisol production after FSK treatment (n=6; p≤ 0.0001). Data represents the mean ± SD Student´s test.

We observed that the induction of oxidative stress, with H_2_O_2_, in H295R adrenal cells caused an increase in ROS production (p < 0.0001) (**Figure 1A**). In response, the mitochondrial activity also was increased under these conditions (p < 0.0001) (**Figure 1B**). This dysregulation of redox homeostasis, caused by oxidative stress, impairs cortisol secretion in adrenal cells (p = 0.0004) (**Figure 1C**).

We also observed the impact of steroidogenesis induction on redox homeostasis. Cortisol increase (p < 0.0001) (**Figure 1F**), by FSK stimulus, resulted in an increase in ROS production (p = 0.002) (**Figure 1D**) and mitochondrial activity (p = 0.006) (**Figure 1E**).

### NNT knockdown in H295R adrenocortical cells

**Figure 2:**
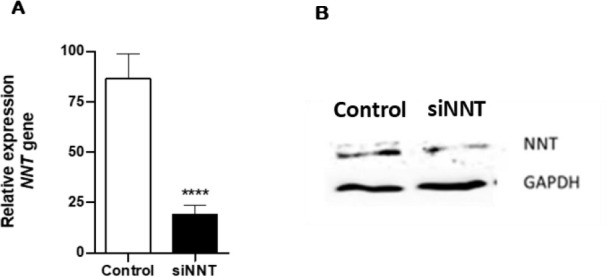
siRNA knock-in of the NNT gene in H295R cells. (**A**) NNT mRNA expression was quantified using real-time qPCR and normalized to GUSB (n=6; p≤ 0.0001). (**B**) Reduced expression of the protein NNT is seen in siNNT after Western blot analysis. Data represents the mean ± SD Student´s test.

Through the application of the RNAi system commercially available, targeting regions of exons 2 to 22 in the NNT gene, we achieved NNT knockdown. Greater than 78% NNT knockdown was established in H295R cells, quantified by real-time quantitative PCR (**Figure 2A**), resulting in a mRNA expression of thereabout 22% in comparison with control H295R. A reduction in NNT protein expression was confirmed by immunoblotting (**Figure 2B**).

### The NNT knockdown in H295R enhances mitochondrial activity while maintaining redox homeostasis

**Figure 3:**
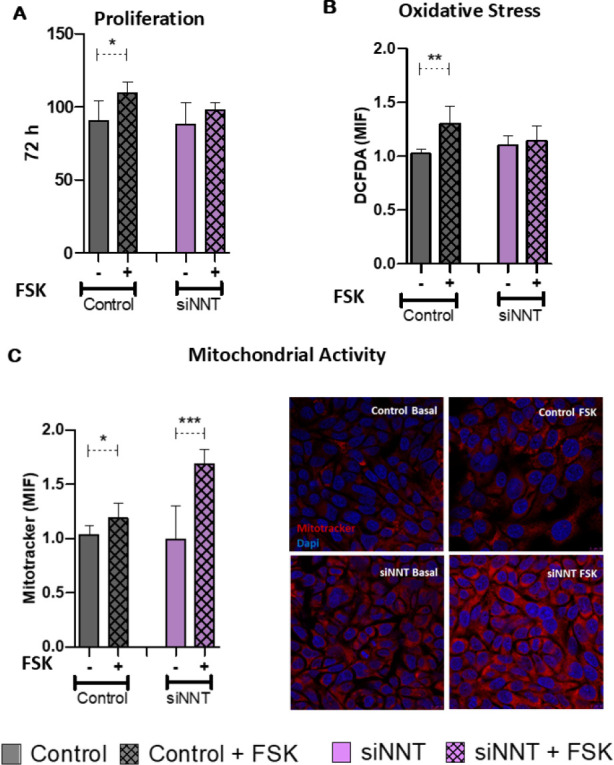
Evaluation of proliferation and mitochondrial function after stimulation of steroidogenesis. (**A**) Cell proliferation analysis indicates an increase in proliferation in control cells (n=6; p≤ 0.02). (**B**) ROS production, quantified with 20,70-dichlorodihydrofluorescein diacetate (DCFDA), in the control cells, who presented increased ROS production after steroidogenesis induced with forskolin (FSK) (n=6; p≤=0.02). (**C**) Quantitative staining with MitoTracker Red showing increased mitochondrial activity in control (n=6; p≤ 0.03) and siNNT cells (n=6; p= 0.0006). Representative image of mitochondrial activity increase in control and siNNT cells.

The number of cells verified after 72 hours of culture, via cell titer assay, showed an increase in control H295R cell proliferation under FSK stimulus (p < 0.002). Despite that, NNT knockdown H295R cells didn’t reveal an increase in proliferation (**Figure 3A**).

Under the same conditions, the oxidative stress profile was verified through DCFDA stain. Control cells showed an enhancement in ROS formation, under steroidogenesis induction conditions (p < 0.02). H295R NNT knockdown cells didn’t reveal ROS increase under the same condition (**Figure 3B**).

Mitochondrial activity was evaluated with the color stain MitoTracker Deep Red FM. Control H295R cells presented a higher absorption of MitoTracker signaling for an increase in mitochondrial activity under FSK stimulus condition (p < 0.003). NNT knockdown cells have shown a greater increase in mitochondrial activity when submitted to steroidogenesis induction (p = 0.006) (**Figure 3C**).

### NNT knockdown in H295R affects cortisol levels without compromising key steroidogenic components

**Figure 4:**
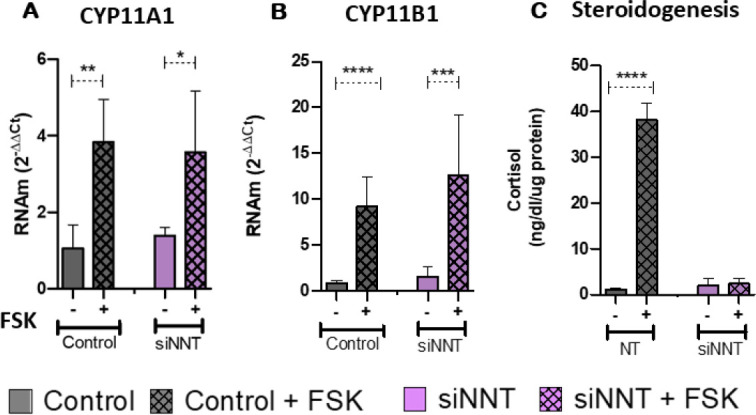
Evaluation of adrenal steroidogenesis. (**A**) Control cells and cells with reduced NNT show increased expression of the CYP11A1 gene (n=6; p=0.001 and p=0.03). (**B**) Control cells and cells with reduced NNT show increased expression of the CYP11B1 gene (n=6; p≤ 0.0001 and p=0.0006). (**C**) Control cells show an increase in cortisol production after stimulation with forskolin (FSK) (n=6; p≤ 0.0001).

After real-time quantitative PCR, we observed that NNT knockdown didn’t alter P450c11α (*CYP11A1*) or P450c11β (*CYP11B1*) transcription pattern under plain medium or steroidogenesis stimulus conditions. The induction of steroidogenesis, through FSK treatment, showed an increase in *CYP11A1* and *CYP11B1* mRNA expression in control (p = 0.001; p < 0.0001) and NNT knockdown (p = 0.03; p = 0.0006) H295R cells (**Figures 4A and 4B**).

FSK treatment in control H295R cells significantly increased the cortisol secretion (p < 0.0001). Nevertheless, NNT knockdown deeply impaired the cortisol secretion in H295R cells under steroidogenic challenge (**Figure 4C**).

### NNT knockdown in H295R alters MAPK8 expression

**Figure 5:**
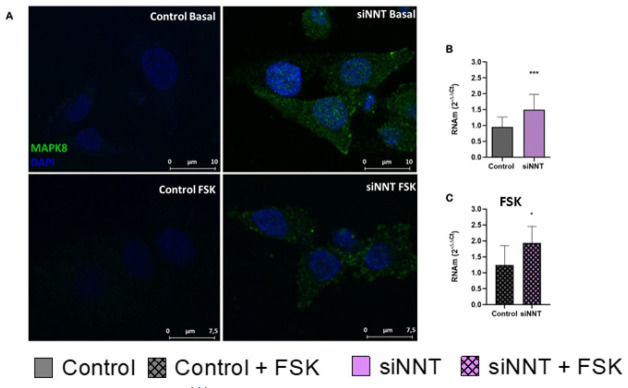
NNT knockdown alters MAPK8 expression. (**A**) Representative figure of MAPK8 protein expression, through immunofluorescence. (**B**) NNT knockdown increases MAPK8 relative gene expression under regular condition (n=6; p=0.008) (**C**) and steroidogenesis stimulus with forskolin (FSK) (n=6; p=0.03).

NNT knockdown increases *MAPK8* relative gene expression, when compared with control cells, under basal (p = 0.008) and steroidogenesis stimulus (p = 0.03) (**Figures 5B and 5C**). We also observed MAPK8 protein expression via immunofluorescence. The green fluorescence is intensified in H295R NNT knockdown cells (**Figure 5)**.

## DISCUSSION

Changes in antioxidant defense mechanisms can lead to the accumulation of ROS. Disruption of the balance of these species leads to a process known as oxidative stress, which can compromise cellular functions and result in damage and death ^(14)^.

Steroidogenesis significantly contributes to mitochondrial ROS production. These species are generated in the process of electron transfers by the P450 system. To prevent the formation of oxidative stress, steroidogenic tissues are supplied with high levels of enzymatic and non-enzymatic antioxidants. Changes in antioxidant defense mechanisms may compromise steroidogenesis ^(15)^.

Studies of oxidative stress induction, on steroidogenic Leydig cell line (MA-10) have shown inhibition in progesterone production ^(3,16)^. In accordance with the literature, we observed that adrenal cells challenged by hydrogen peroxide stimulus showed an increase in ROS production as well as in mitochondrial activity. The creation of a stressor environment triggered an important decrease in cortisol production, resulting in a compromise of steroidogenesis in this cell line. The oxidative stress impact on steroidogenesis, in adrenal cells, goes both ways. Our data showed that the increase on steroidogenesis via forskolin stimulus leads to an increase in mitochondrial activity and ROS production in response to the stressor challenge.

NNT protein plays a massive role in ROS elimination and the maintenance of cellular homeostasis. Its primary function is to maintain high concentrations of NADPH in the intramitochondrial environment, responsible for GSH to GSSG regeneration and increasing the GSH/GSSG ratio during the ROS degradation process ^(6)^.

Although the importance of NNT protein in adrenal steroidogenesis has been widely known ^(6,10)^, there is still a lack of knowledge to be enlightened here. This study analyzed the in vitro adrenocortical cell line H295R to evaluate the impact of NNT knockdown on adrenal steroidogenesis, ROS production, and mitochondrial activity.

Through silencing with siRNA, our experiments were able to transiently reduce the expression of the NNT protein in the H295R cell line. Under these conditions, the cells were able to maintain the normal functioning of antioxidant defense mechanisms, as we can observe in the analysis of ROS formation and mitochondrial function.

Studies of chronic reduction in NNT expression, made with CRISPR-Cas9 or Short hairpin permanent depletion of expression models, showed impairment in mitochondrial functions leading to oxidative stress ^(8,9)^. In contrast, our work evaluated the acute effects of NNT knockdown induced by iRNA, which results in a transient reduction in expression. Thus, the impairment of mitochondrial function appears to be related to the duration of exposure to this stressful situation. Furthermore, the introduction of a new stressor, induced by FSK to stimulate steroidogenesis, showed that cells deficient in NNT must increase mitochondrial demand to maintain cellular homeostasis.

As expected, we found that FSK in control cells stimulates steroidogenesis, resulting in increased cortisol secretion and expression of CYP11A1 and CYP11B1 genes. When evaluating the response to this stimulus in cells with NNT knockdown, we noticed a distinct response. Although the expression of the CYP11A1 and CYP11B1 genes is maintained, they do not result in increased cortisol secretion. This observation indicates that damage to the NNT protein impacts cortisol secretion. These data show that, faced with a partial and temporary decrease in the function of the NNT protein, cells activate a predilection mechanism, blocking steroidogenesis to moderate the increase in ROS and maintain cellular homeostasis.

Oxidative stress stimulates the activation of several intracellular pathways, including the stress-activated protein kinase signaling pathway, c-Jun-N-terminal (SAP/JNK). In this pathway, two dual-specificity kinases, MAP2K4 and MAP2K7, phosphorylate and activate the MAPK8 protein. MAPK8 promotes cell apoptosis, decreased gap junctions (GAP) between cells, fibrosis, increased expression of genes for hypertrophy, and mitochondrial dysfunction. During this process, there is an increase in the activation of the MAPK8 protein located in the cytosol and its translocation to the inner mitochondrial membrane, which suggests an interconnection between the cytosol and mitochondria for the maintenance of redox balance ^(11,17)^.

In a study using adrenal pheochromocytoma cells from PC12 rats, silenced NNT gene promoted a reduction in intracellular NADPH levels, the GSH/GSSG ratio, and an increase in H_2_O_2_ levels, culminating in an increase in the oxidative state. The reduction of NNT expression in these cells also resulted in an increase in the expression of phosphorylated MAPK8 ^(11)^. This fact was also observed in the present study; as we reduced NNT expression in H295R cell line, an increase in gene and protein expression of MAPK8 was observed. In our study, the cells did not show dysregulation in redox balance, which suggests that the activation of the MAPK8 pathway may be serving as a compensatory pathway in these cells, allowing them to maintain redox balance at the expense of cortisol secretion.

Several studies using animal models have demonstrated the relationship between oxidative stress, autophagy, and multiple signaling pathways in maintaining cellular homeostasis. ROS generation has been associated with the activation of MAPK pathways, which can influence mitochondrial integrity, as well as the PI3K/AKT/mTOR signaling cascade, which regulates autophagy and cell survival. These findings support our observations, in which oxidative stress induced by NNT deficiency may trigger compensatory activation of MAPK8, helping to preserve mitochondrial homeostasis and partially sustain steroidogenic function. Overall, these results reinforce the idea that redox imbalance engages multiple signaling pathways to maintain cell survival and function, and they highlight potential targets for future investigation ^(18,19)^.

These findings regarding the impairment of steroidogenesis with the protein knockdown of NNT are consistent with what is observed in the clinic in patients with adrenal insufficiency. In recent years, mutations in the NNT gene have been associated with type 4 glucocorticoid deficiency with or without mineralocorticoid deficiency phenotype ^(6,8,20,21)^.

The relationship between NNT and oxidative stress is well established in the literature; however, our study provides important contributions by demonstrating how stress conditions can further compromise adrenal redox homeostasis and reveal compensatory mechanisms, such as MAPK8 activation, that help partially preserve mitochondrial and steroidogenic function. Despite the new insights provided by this study, some limitations should be acknowledged. Notably, the reduction of NNT was partial, which may not fully reproduce complete enzyme deficiency. Furthermore, the analysis was restricted to total MAPK8 protein expression, without evaluating its phosphorylated or activated forms, limiting conclusions regarding MAPK8 functional regulation and compensatory mechanisms. Future investigations could address these gaps by including analyses of MAPK8 phosphorylation, direct assessment of the activity of tricarboxylic acid (TCA) cycle enzymes, or evaluation of mitochondrial complex subunit expression, which could offer a broader view of cellular energy metabolism. These observations highlight important directions for future research and reinforce the relevance of this study in elucidating the role of NNT and compensatory mechanisms in adrenal redox homeostasis and steroidogenesis.

**Figure 6:**
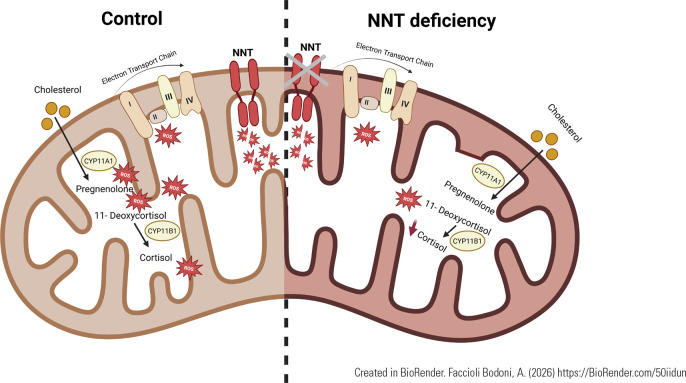
Representative figure of mitochondrial activity. In control cells, we observe that electron transport through the mitochondrial chain and the release of ROS during steroidogenesis are depleted via the NNT intermediate. In NNT deficiency, our data suggest a decrease in cortisol production to maintain cellular homeostasis.

The NNT protein is localized to the inner mitochondrial membrane and plays a crucial role in maintaining cellular redox balance. Under physiological conditions, ATP production by the mitochondrial respiratory chain partially reduces molecular oxygen, generating reactive oxygen species (ROS). In adrenal cells, steroidogenesis further contributes to mitochondrial ROS production, particularly during the conversion of pregnenolone to 11-deoxycortisol, thereby increasing the oxidative burden within the mitochondrial matrix. In this context, NNT is essential for preserving mitochondrial function and cellular homeostasis by sustaining NADPH availability. In NNT-deficient cells, ROS generated by both oxidative phosphorylation and steroidogenic activity accumulate over time due to impaired NADPH production. Consequently, steroidogenesis is attenuated, likely as a compensatory mechanism to limit oxidative stress and prioritize ATP generation through mitochondrial respiration (**Figure 6**) ^(1,14)^.

Created in BioRender. Faccioli Bodoni, A. (2026) https://BioRender.com/50iidun

The genotype-phenotype relationship in NNT deficiency remains poorly defined, reflecting the marked clinical heterogeneity observed in affected individuals. Although initially described as a cause of familial isolated glucocorticoid deficiency, clinical evidence has shown that patients harboring pathogenic variants in NNT may present a broader spectrum of adrenal impairment, including mineralocorticoid deficiency. In addition, in recent years, alterations in gonadal steroidogenesis and even disorders of sex development have been reported, indicating that multiple steroidogenic axes may be affected ^(22)^. Our experimental data suggest a possible mechanism underlying this clinical variability by demonstrating that NNT-deficient cells can maintain steroidogenesis and cellular homeostasis for a period, with functional impairment becoming evident only after chronic exposure to NNT deficiency. This delayed phenotypic manifestation suggests the involvement of compensatory mechanisms, such as the activation of oxidative stress-responsive pathways, including MAPK8, which may transiently sustain steroidogenic function. Consistently, patients with NNT deficiency typically do not present with clinical manifestations in the first days of life, with diagnosis often established after the first year of life or at later ages, reinforcing the concept that progressive failure of these adaptive mechanisms contributes to the observed phenotypic variability ^(22)^.

In summary, NNT-deficient adrenocortical cells can maintain mitochondrial homeostasis and cortisol secretion in basal conditions. However, under stress, these cells seem to maintain the redox balance at the expense of impaired steroidogenesis. MAPK8 pathway activation appears to compensate for NNT deficiency.

## Data Availability

datasets related to this article will be available upon request to the corresponding author.
